# Nasal sarcoidosis

**DOI:** 10.11604/pamj.2021.40.134.32076

**Published:** 2021-11-03

**Authors:** Fadwa Mekouar, Mohamed Elqatni

**Affiliations:** 1 Internal Medicine B, Mohammed V Military Teaching Hospital, Sidi Mohamed Ben Abdellah University, Fes, Morocco

**Keywords:** Sarcoidosis, granulomatous disease, diagnosis

## Image in medicine

A 19-year-old woman with a two years history of dragging oto-rhino-laryngeal infections, purulent rhinorrhea and sinusitis, polyarthralgia, and an episode of granulomatous uveitis. Clinical examination showed destruction of nasal wing, hepatomegaly and splenomegaly. A rounded lesion with atrophic center in the forearm. Laboratory tests revealed upper limit calcemia, the angiotensin-converting enzyme was high 323 UI/L. Nasal biopsy revealed non-caseating epithelioid-cell granulomas. Differential diagnosis includes granulomatous disease of the nose such as leprosy, tuberculosis and Wegener’s granulomatosis. Special staining for typical mycobacterium and lepra bacilli were negative. The polymerase chain reaction for mycobacterium tuberculosis was negative. There was no renal involvement and the cytoplasmic antineutrophil cytoplasmic antibody (cANCA/PR3/ANCA) was negative. The diagnosis of nasal sarcoidosis was retained. The patient was treated with prednisone and azathioprine with favorable outcome.

**Figure 1:**
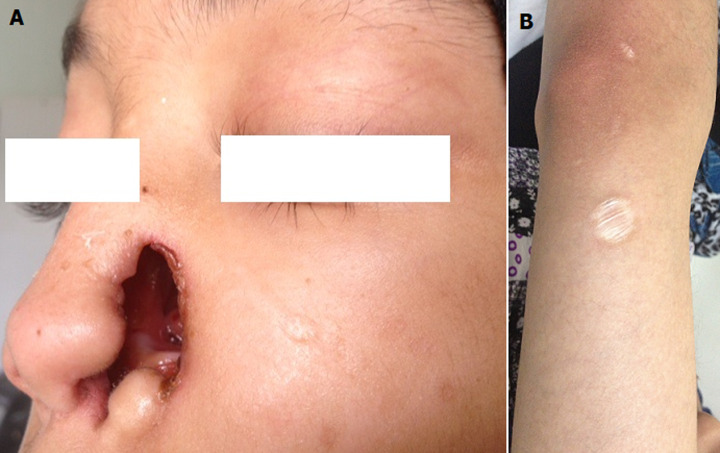
:A) destruction of nasal wing, B) rounded lesion with atrophic center in the forearm

